# Supplementary material for: Mutation in FBXO32 causes dilated cardiomyopathy through up-regulation of ER-stress mediated apoptosis
Source: Commun Biol. 2021 Jul 16;4:884. doi: 10.1038/s42003-021-02391-9 (PMC8285540; doi:10.1038/s42003-021-02391-9)
Supplement: Supplementary file 2 — Supplementary Information [file 42003_2021_2391_MOESM2_ESM.pdf]

Supplementary Figures

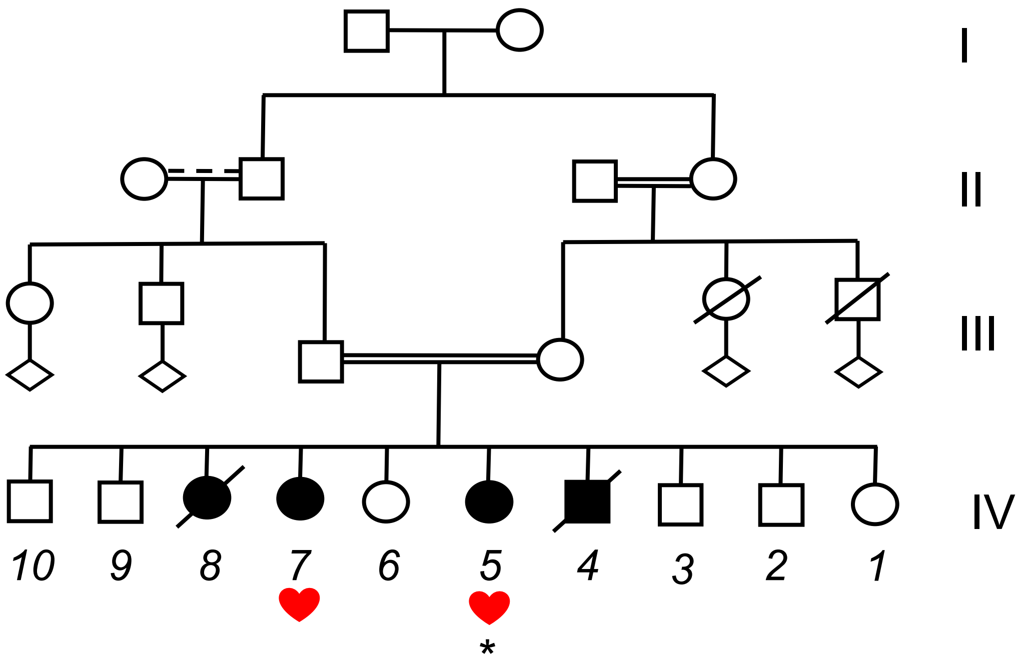

Supplementary Figure 1 : Pedigree of the consangineous Family A showing the four children carrying the homozygous FBXO32-G243R mutation and that develop DCM. Black squares (males) or circles (females) indicate the affected patients and the white symbols show the unaffected members. The hearts collected from patients IV.5 and IV.7 are shown with a heart symbol. Exome sequencing was performed on patient IV.5 marked with an asterisk.

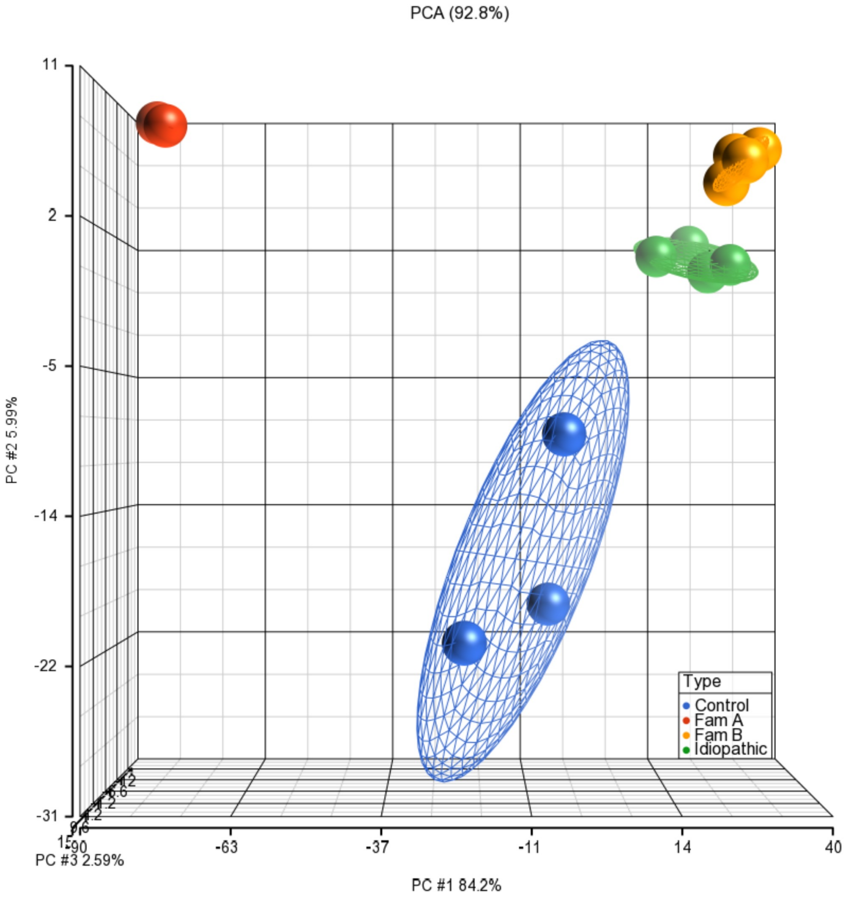

Supplementary Figure 2 : Principal component analysis of Affymetrix microarray data. An unsupervised principle components analysis (PCA) of the microarray data, which contained about 91.6% of the variance in the data matrix, clearly distinguished individuals in four clusters: Family A (patient with the *FBXO32* mutation), Family B (patient carrying another mutation from an unrelated family), control hearts and idiopathic dilated hearts. The blue spheres represent the control hearts, red spheres are technical replicates for Family A, yellow spheres are technical replicates for Family B and green spheres for the idiopathic dilated hearts.

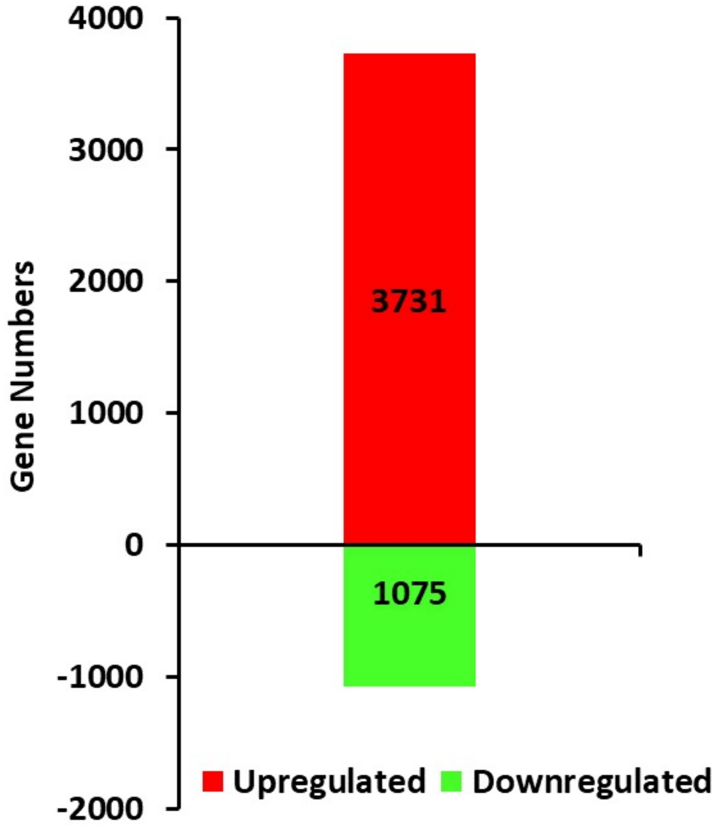

Supplementary Figure 3 : **Total number of differentially regulated genes in patient heart carrying the *FBXO32* mutation.** Results from the transcriptional profiling show that among the 4806 mRNA differentially regulated in the *FBXO32* mutant heart, 3731 were significantly up-regulated and 1075 were significantly down-regulated. Red indicates up-regulated and green shows the down-regulated genes.

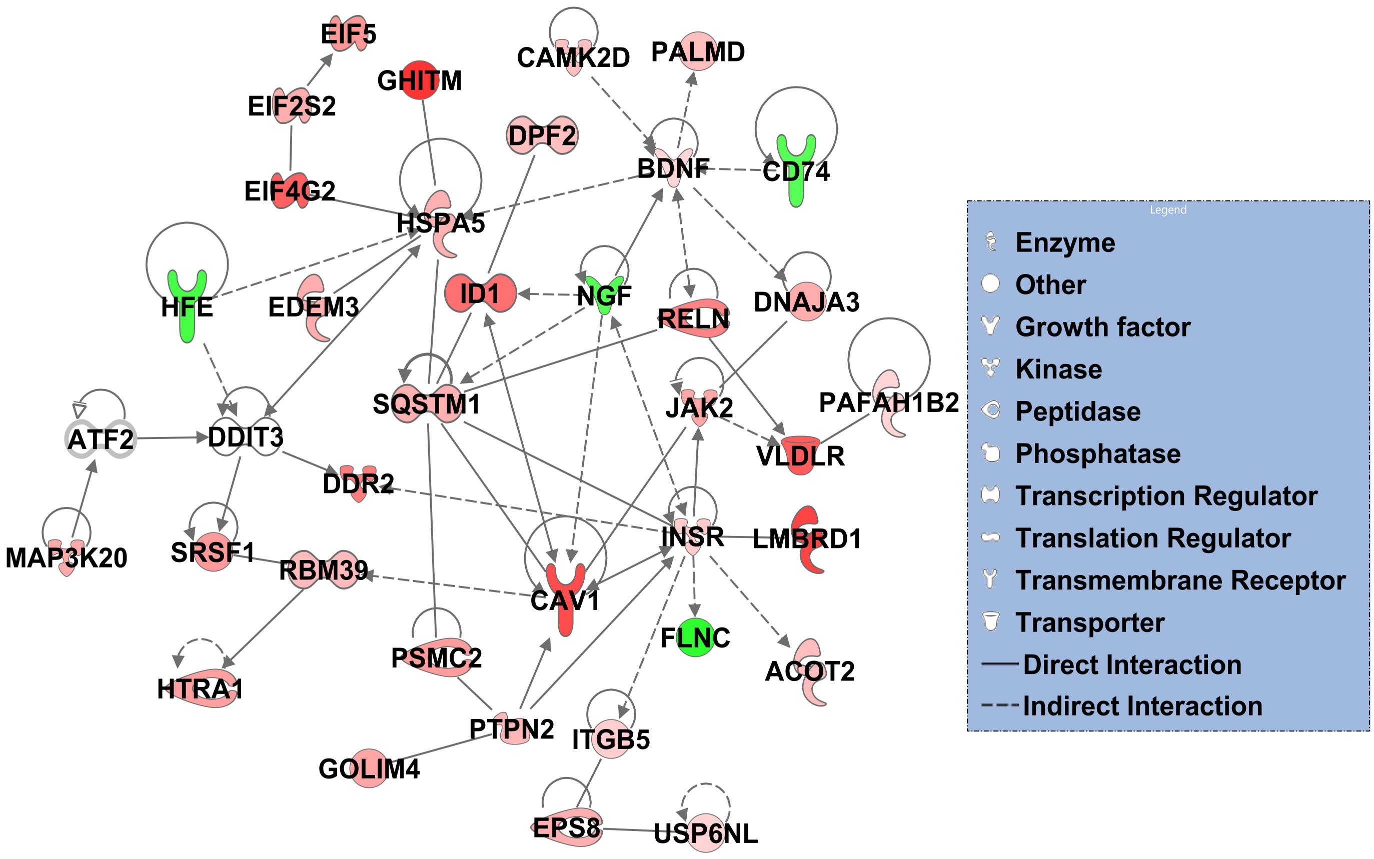

Supplementary Figure 4 : Network interaction of CHOP target genes from the microarray data enriched in *FBXO32* mutant hearts. Nodes represent genes, with their shape representing the functional class of the gene product, and edges indicate biological relationship between the nodes. Green (red) indicates down- (up-) regulated genes in patient compared to controls. The color intensity is correlated with the fold change.

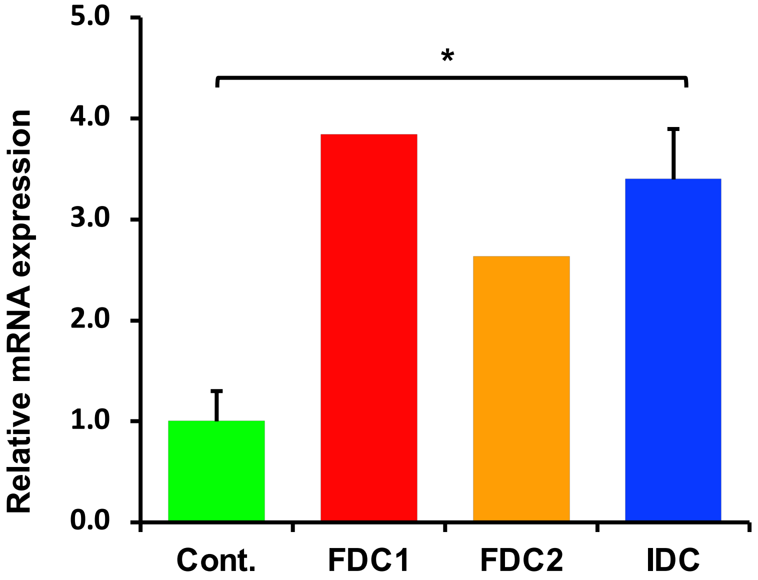

Supplementary Figure 5 : ATF2 mRNA expression in the different groups of hearts. There is no significant differences in ATF2-mRNA level between IDC and FDC patient hearts. The mRNA levels were measured by quantitative RT-PCR from three control hearts, *FBXO32* mutant hearts (Familial FDC1 and FDC2, patients IV.5 and IV.7 respectively) and three IDC hearts. ATF2 mRNA expression was normalized to mRNA levels of the housekeeping gene GAPDH. The statistical significance was determined using a Student's t test. * $p \leq 0.049$.

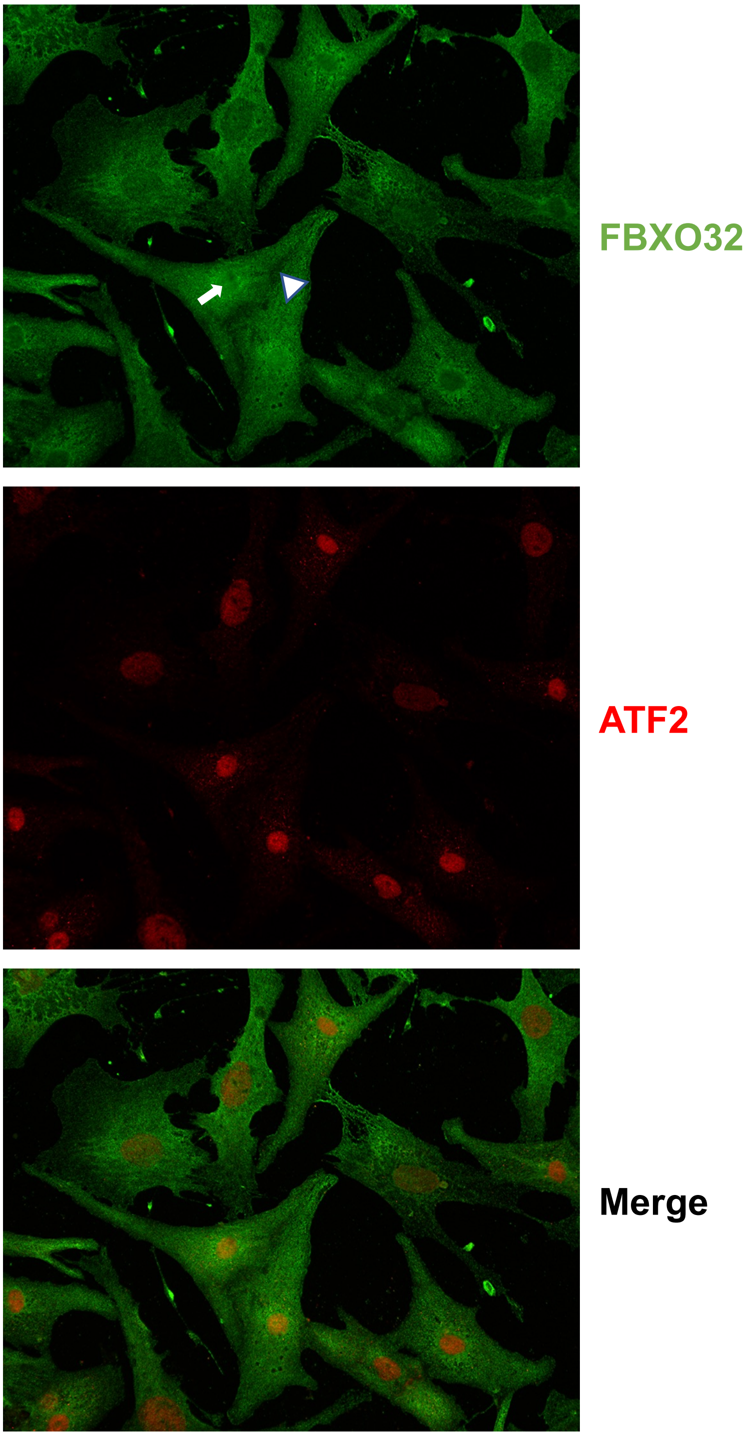

Supplementary Figure 6 : FBXO32 co-localizes with ATF2 in NRVM. Confocal immunofluorescence in neonatal rat ventricular myocytes (NRVM) isolated from 2 days-old pups and maintained in culture in the presence of 5% fetal bovine serum. After transduction with a lentivirus expressing WT-FBXO32, indirect immunofluorescence was performed using anti-FBXO32 and ATF2 antibodies. Signals were visualized using confocal microscopy (40x magnification). FBXO32 is expressed in the cytoplasm (arrowhead) and in the nucleus (arrow). Merge signals show co-localization of FBXO32 with ATF2. Green: FBXO32; Red: ATF2. Merge signals are shown.

Original Western blots

Shown in Figure 2 a

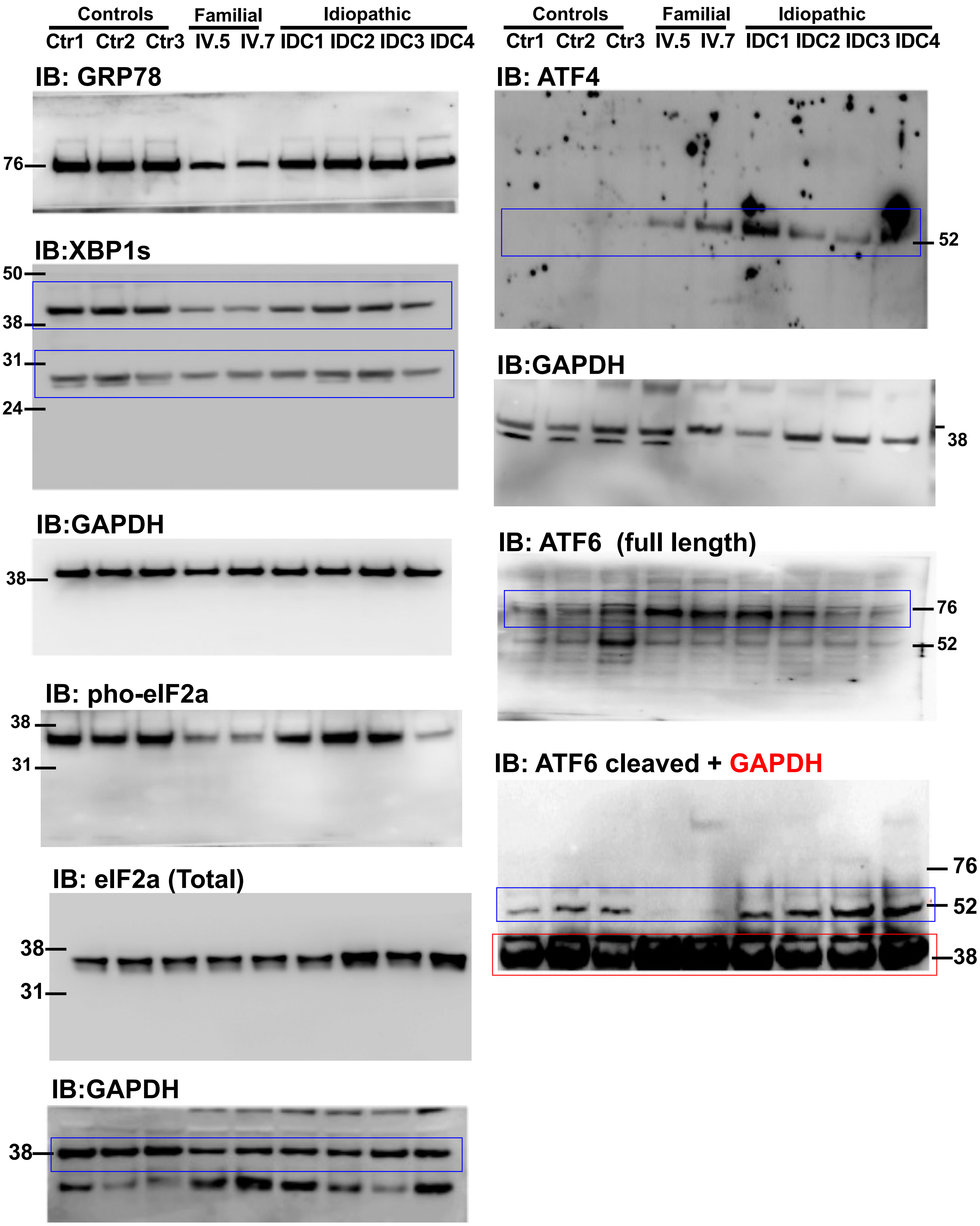

Shown in Figure 2 a

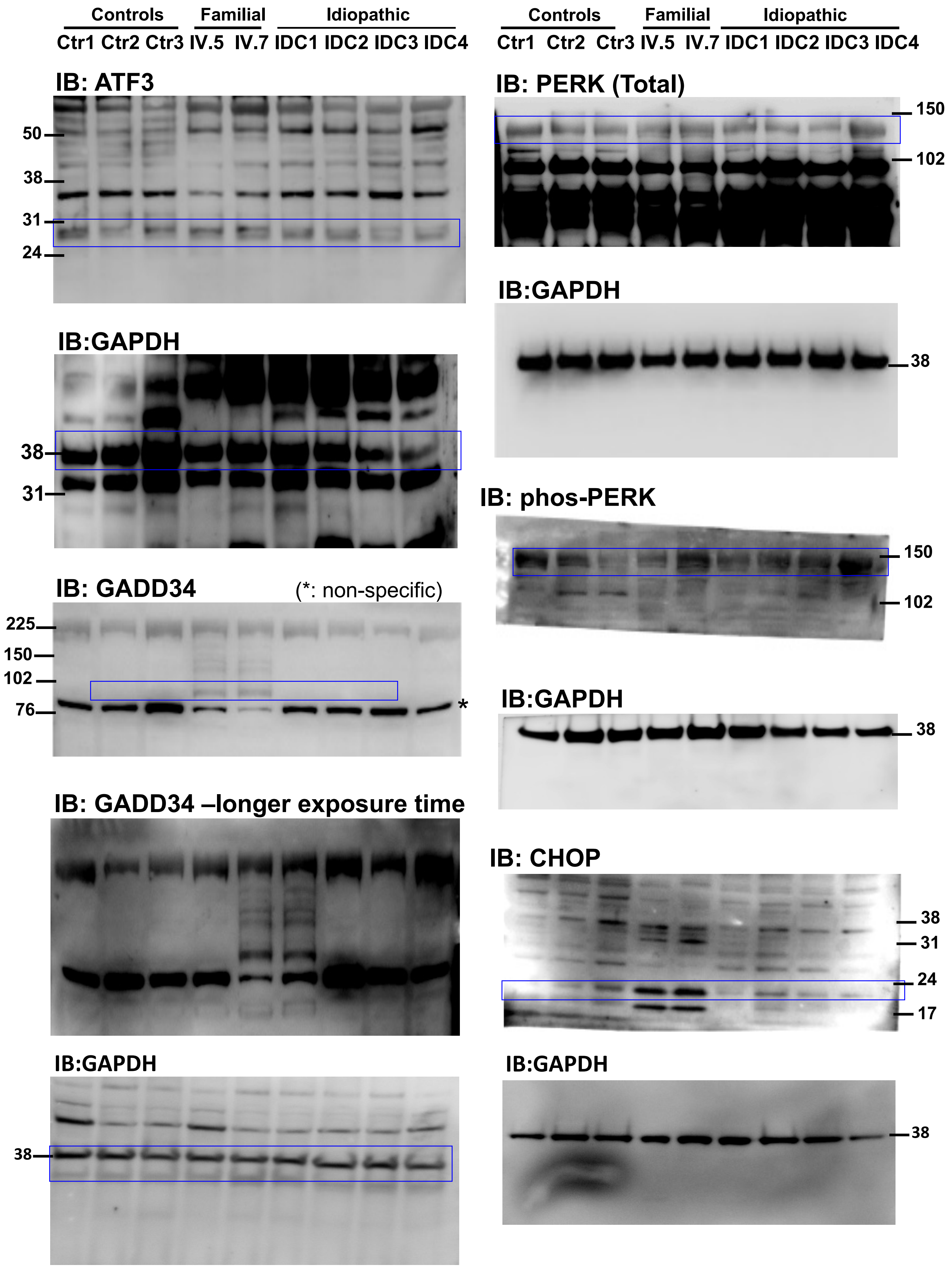

Shown in Figure 3 a

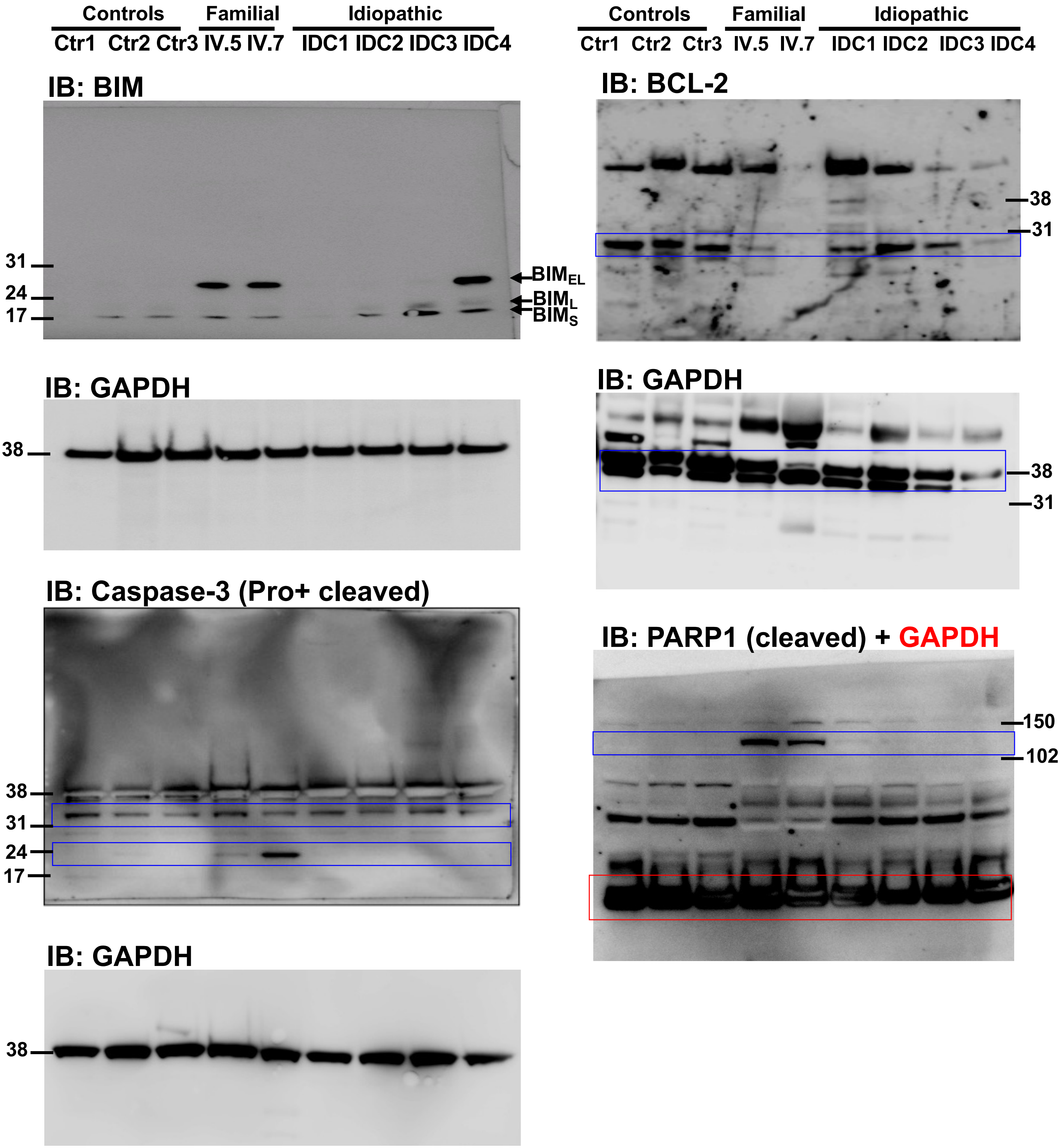

Shown in Figure 4 b

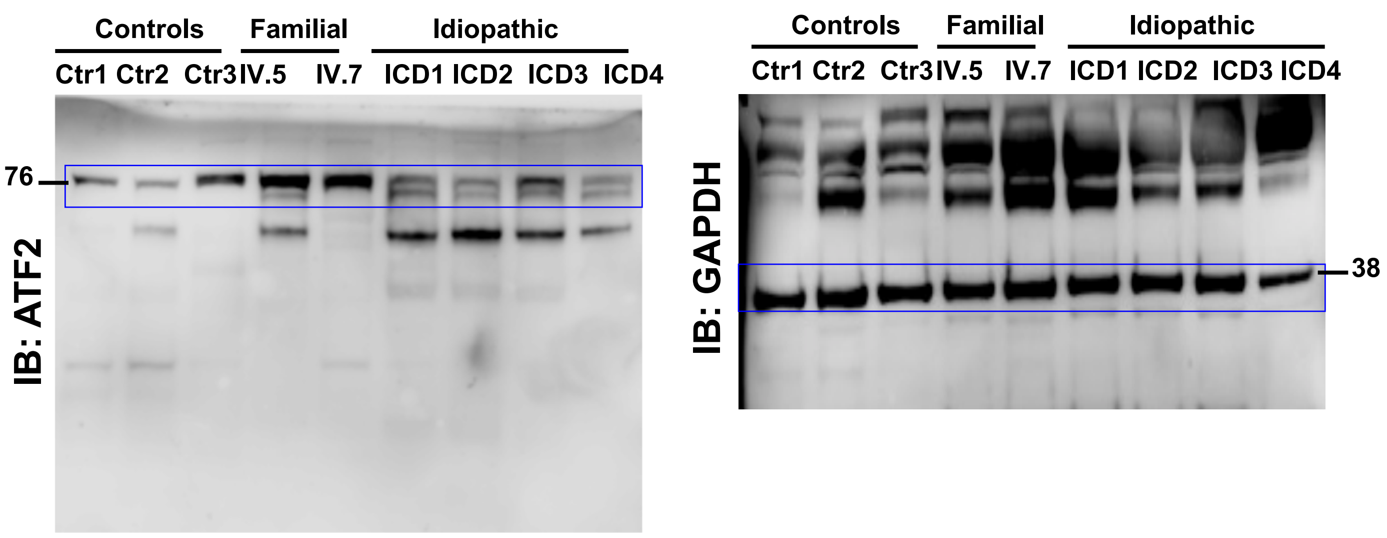

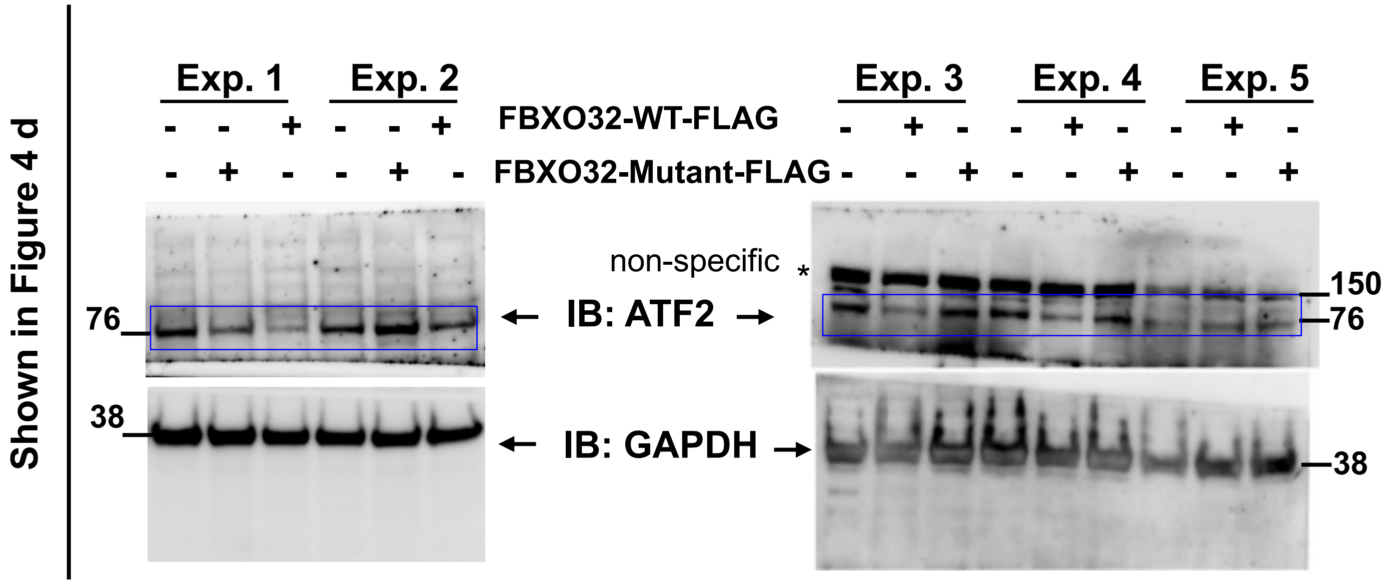

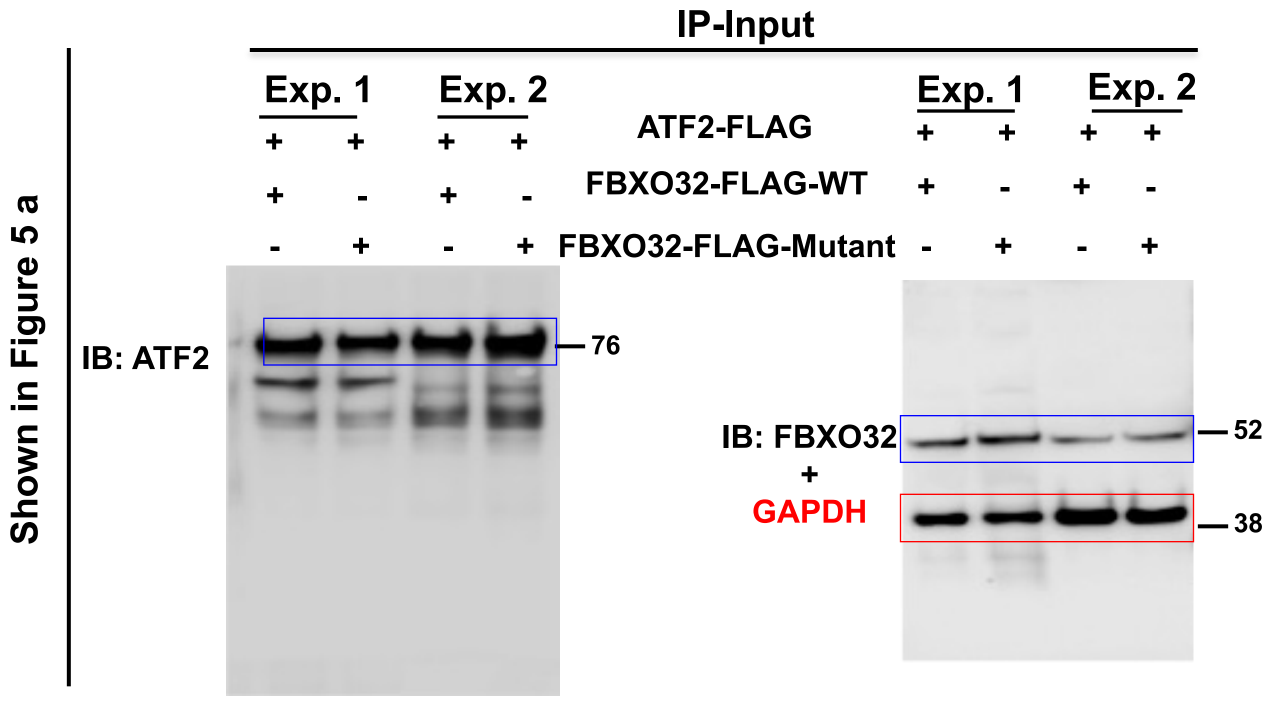

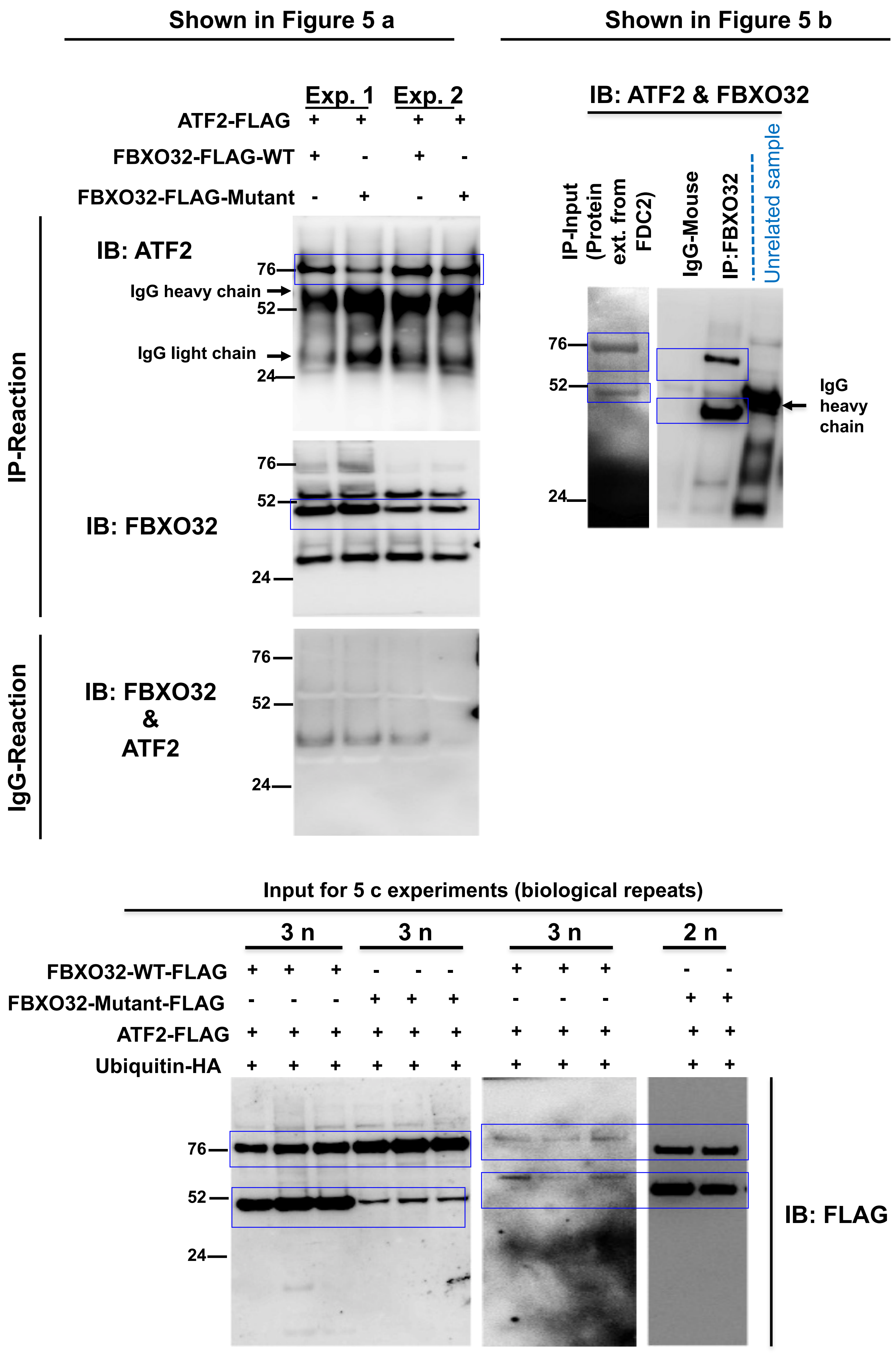
Shown in Figure 5 a
Exp. 1
Exp. 2
ATF2-FLAG + + + +
FBXO32-FLAG-WT + - + -
FBXO32-FLAG-Mutant - + - +
IP-Reaction
IB: ATF2
76
IgG heavy chain
52
IgG light chain
24
IB: FBXO32
76
52
24
IgG-Reaction
IB: FBXO32 & ATF2
76
52
24
Shown in Figure 5 b
IB: ATF2 & FBXO32
IP-Input (Protein ext. from FDC2)
IgG-Mouse
IP:FBXO32
Unrelated sample
76
52
24
IgG heavy chain
Input for 5 c experiments (biological repeats)
3 n
3 n
3 n
2 n
FBXO32-WT-FLAG + + + - - - + + + - -
FBXO32-Mutant-FLAG - - - + + + - - - + +
ATF2-FLAG + + + + + + + + + + +
Ubiquitin-HA + + + + + + + + + + +
76
52
24
IB: FLAG

Shown in Figure 5 c

6 experiments representing 6 biological repeats for WT and 5 for mutant

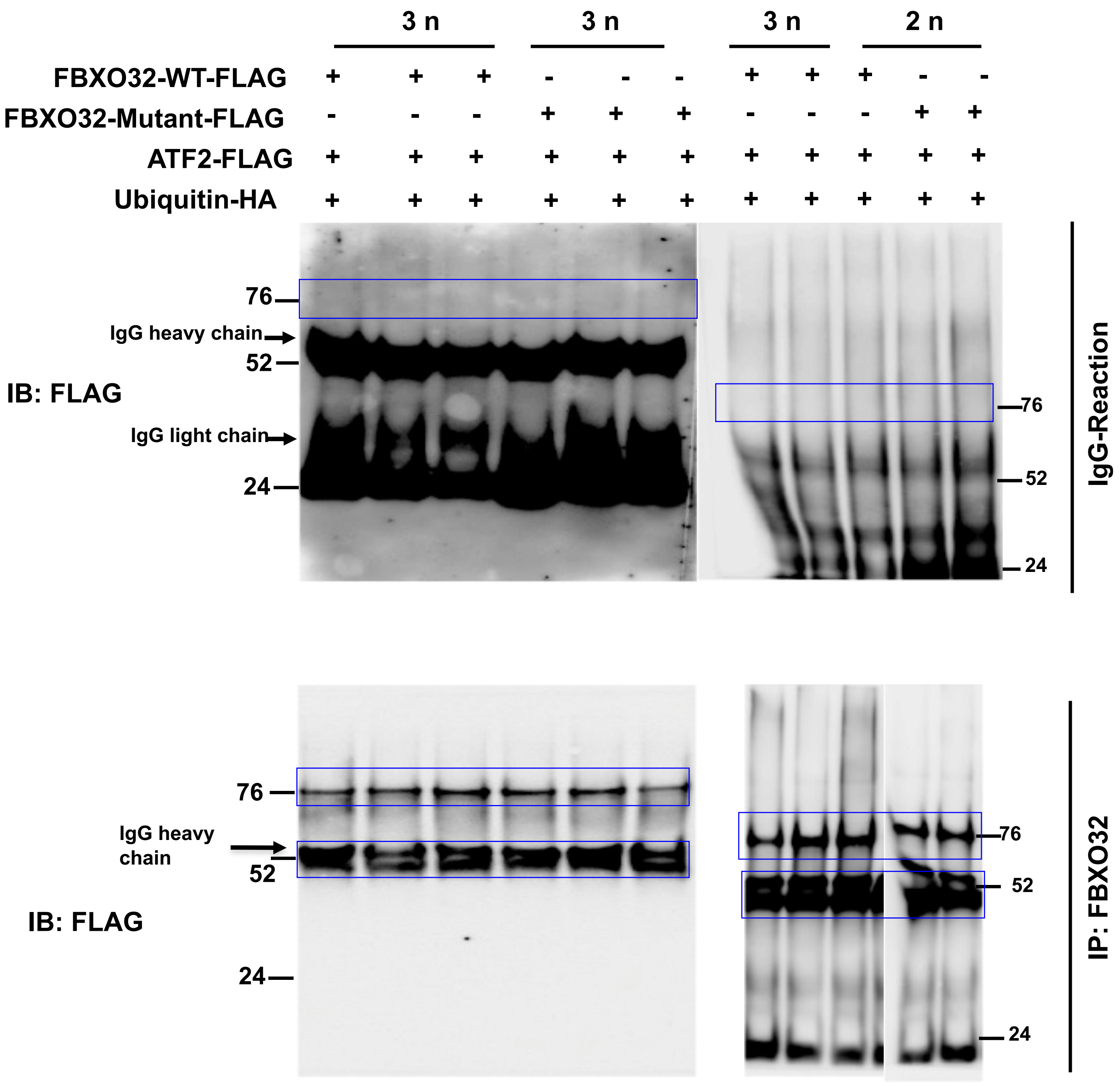

Shown in Figure 5 c

6 experiments representing 6 biological repeats for WT and 5 for mutant

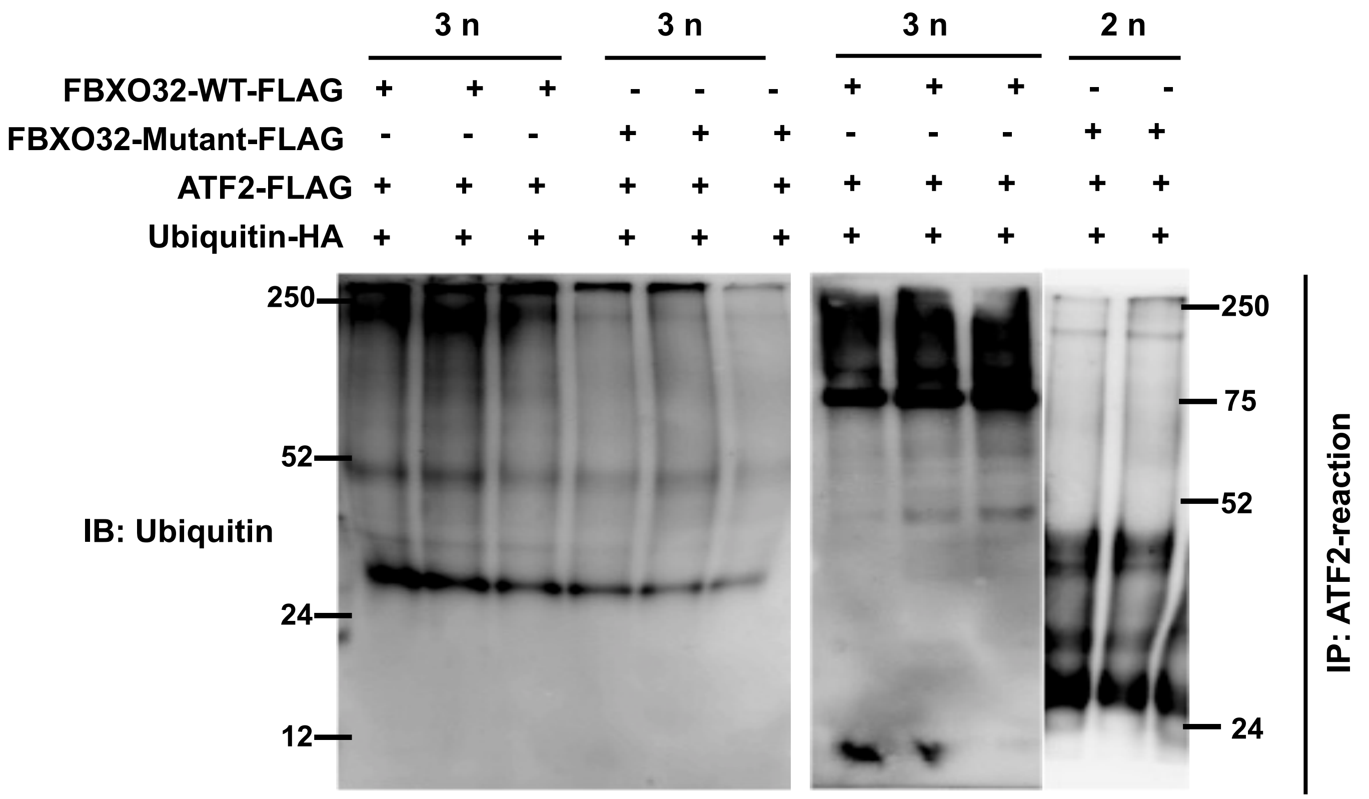

Shown in Figure 6 b

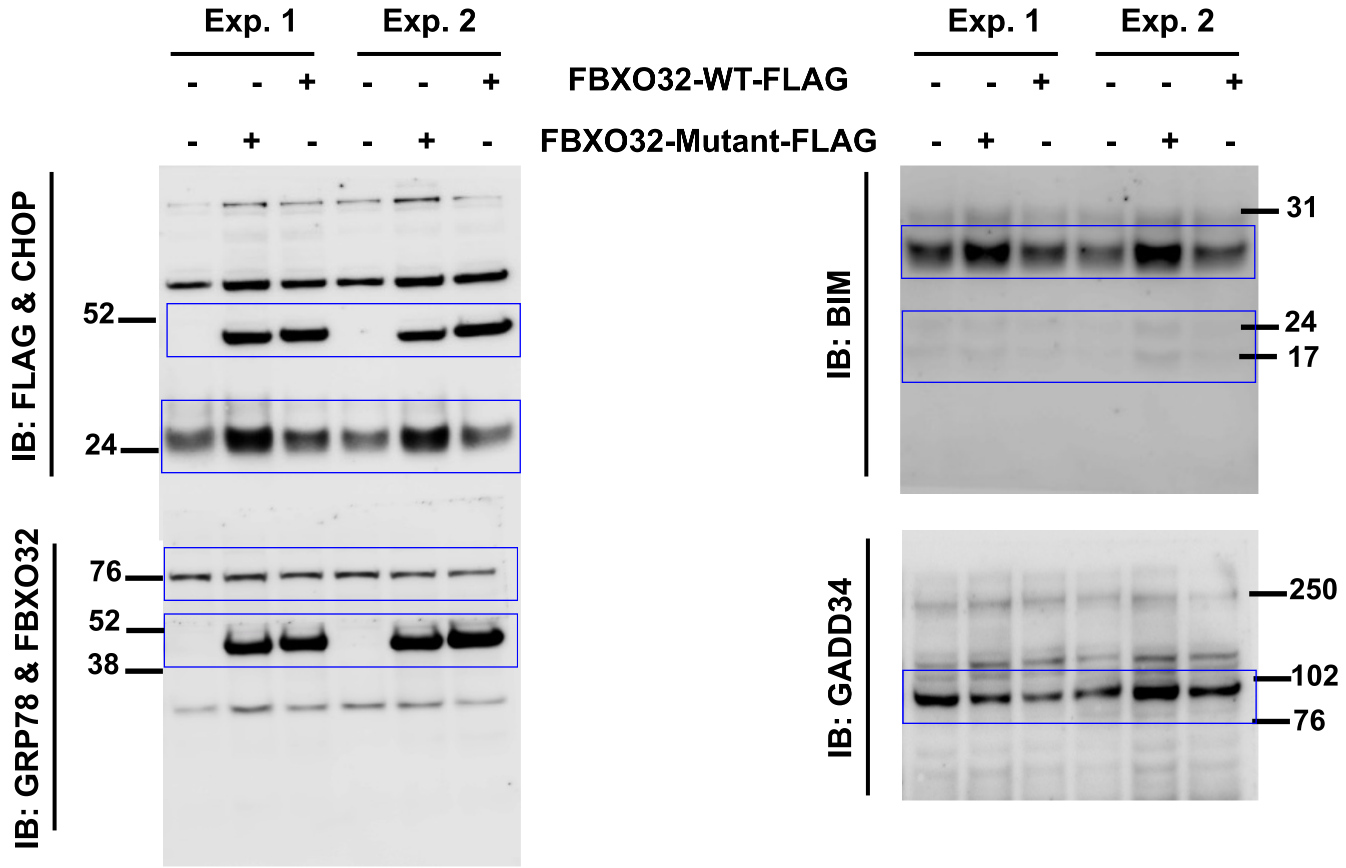

Shown in Figure 6 b

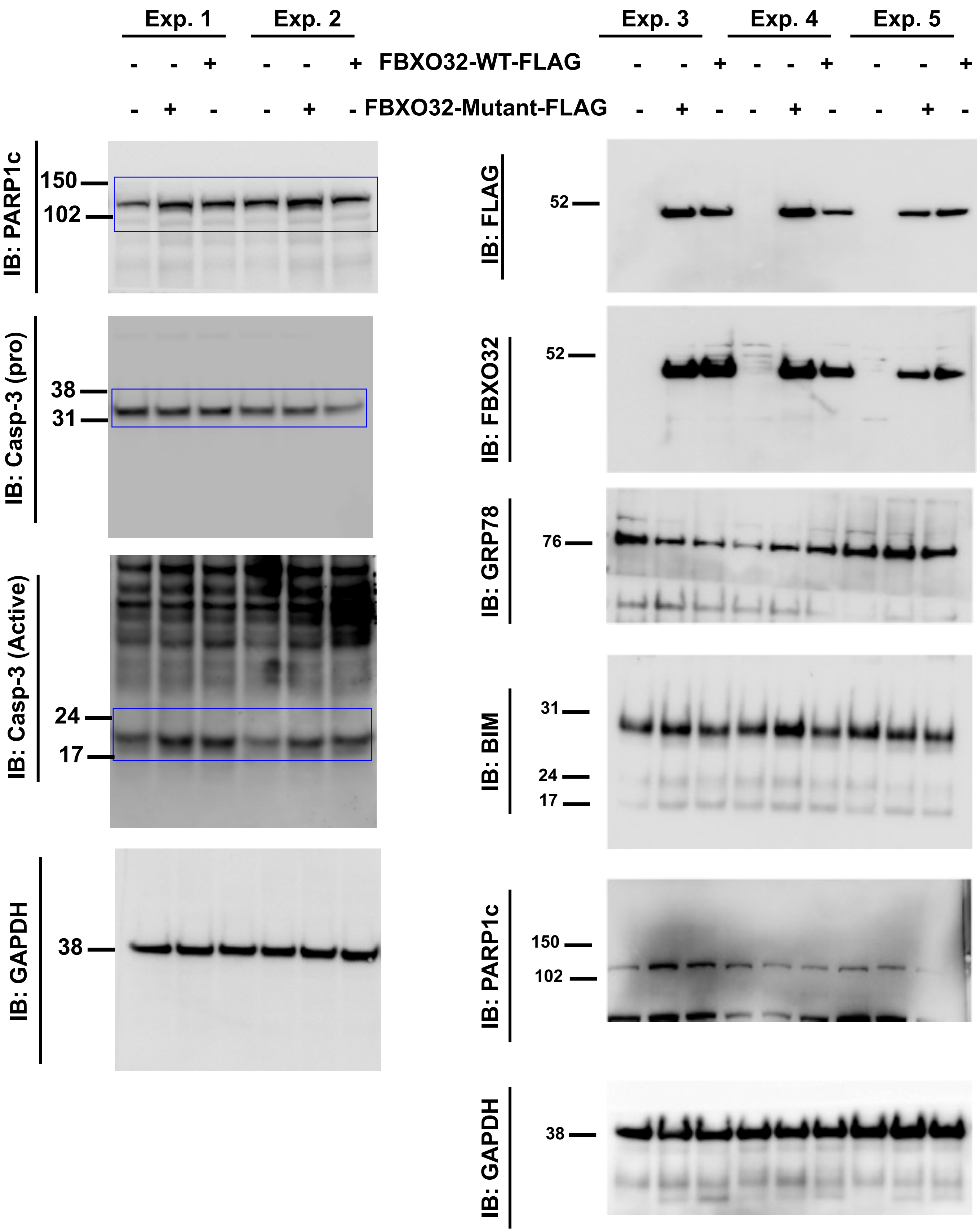

Shown in Figure 6 b

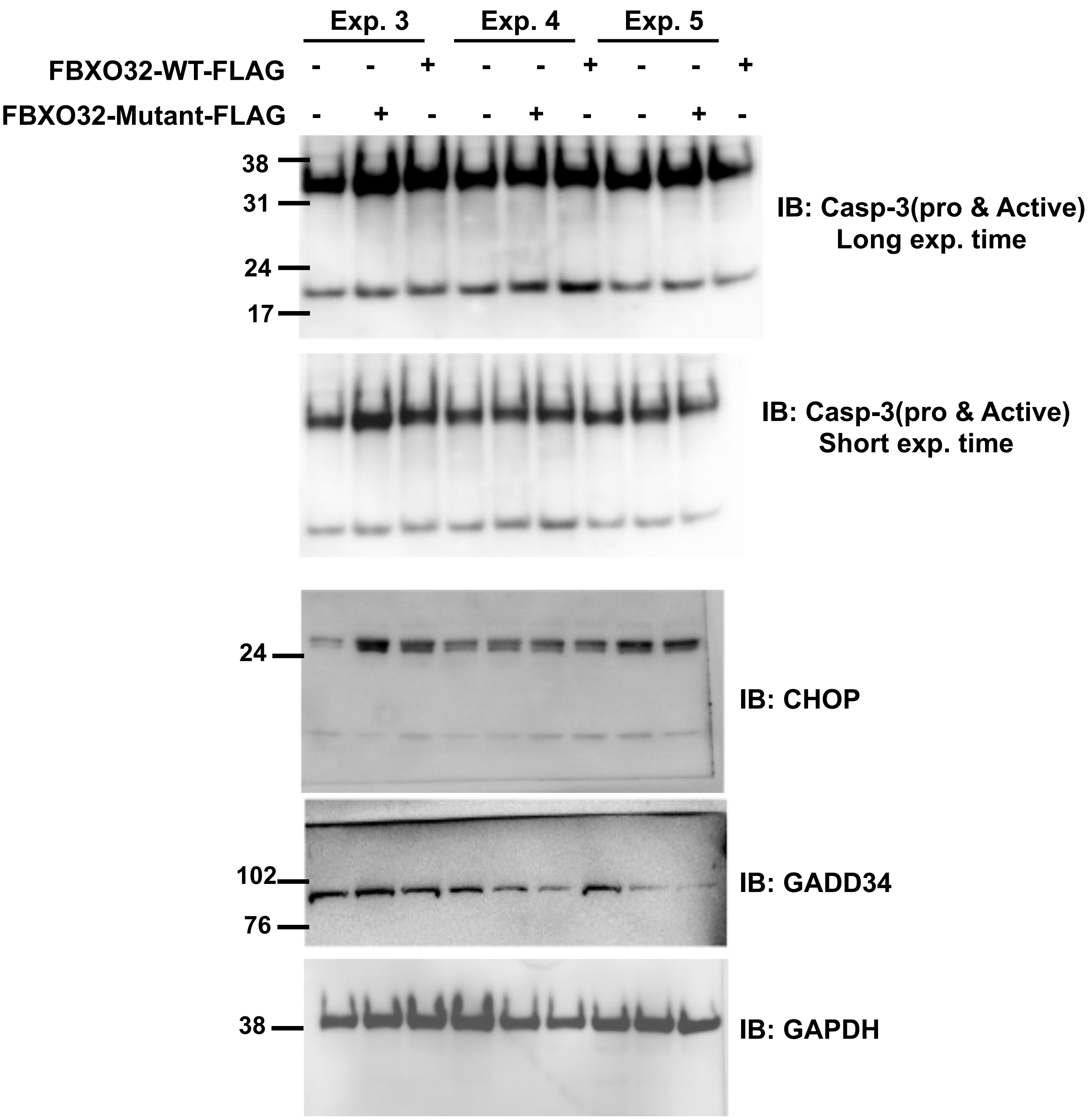

Note for the Original Western blots: Uncropped immunoblots are shown for Fig. 1c, Fig. 2h-i, Fig. 4a, Fig. 4c, Fig. 6b- c, Fig. 7g, Fig. 8b, Supplementary Fig. 2a-b and Supplementary Fig. 4b. Blue squares indicate bands corresponding to the investigated protein. Red labeling and red squares are used when a second primary antibody was used for the same immunoblot.
