# Supplementary material for: Mutation in FBXO32 causes dilated cardiomyopathy through up-regulation of ER-stress mediated apoptosis
Source: Commun Biol. 2021 Jul 16;4:884. doi: 10.1038/s42003-021-02391-9 (PMC8285540; doi:10.1038/s42003-021-02391-9)
Supplement: Supplementary file 3 — Description of Supplementary Files [file 42003_2021_2391_MOESM3_ESM.pdf]

Description of Additional Supplementary Files

File name: Supplementary Data 1
Description: Gene Ontology (GO) enrichment analysis of biological processes associated with significantly dysregulated genes in FBXO32 mutant hearts.

File name: Supplementary Data 2
Description: List of dysregulated genes with roles in the ER stress response in FBXO32 mutant hearts.

File name: Supplementary Data 3
Description: List of CHOP target genes from the microarray data analysis in FBXO32 mutant hearts.

File name: Supplementary Data 4
Description: Network analysis of CHOP target genes in FBXO32 mutant hearts.

File name: Supplementary Data 5
Description: Source data for graphs
